# Caspases as master regulators of programmed cell death: apoptosis, pyroptosis and beyond

**DOI:** 10.1038/s12276-025-01470-9

**Published:** 2025-06-24

**Authors:** So Hee Dho, Minjeong Cho, Wonjin Woo, Seolhee Jeong, Lark Kyun Kim

**Affiliations:** 1 Department of Biomedical Sciences, Graduate School of Medical Science, Brain Korea 21 Project, Gangnam Severance Hospital, Yonsei University College of Medicine, Seoul, Korea; 2 Division of Biology, Pohang University of Science and Technology, Pohang, Korea

**Keywords:** Cell death, Inflammation

## Abstract

Caspases are crucial regulators of programmed cell death, mediating pathways such as apoptosis, pyroptosis, necroptosis and ferroptosis. Their activity is intricately controlled by epigenetic modifications, molecular interactions and post-translational changes, reflecting their central role in cellular homeostasis and disease mechanisms. Dysregulated caspase functions are linked to a wide array of conditions, including cancer, neurodegenerative disorders and inflammatory diseases, establishing their importance as potential therapeutic targets. The roles and regulation of caspases across subcellular compartments and their molecular interactions provide critical insights into the complexity of programmed cell death. Here, this review synthesizes current knowledge on the diverse functions of caspases, offering a comprehensive foundation for exploring innovative therapeutic strategies.

## Caspases control cell death pathways in health and disease

Caspases are special proteins that help control how and when cells die, which is important for keeping our bodies healthy. This study looks at how caspases work in different types of cell death, such as pyroptosis, necroptosis and apoptosis. For example, in pyroptosis, caspases activate proteins called gasdermins that create holes in cell membranes, leading to cell death. In necroptosis, caspases can prevent cell death by breaking down certain proteins. In apoptosis, caspases help dismantle the cell in an orderly way. This study used various methods to understand how caspases interact with other proteins and how they are regulated by changes in DNA and proteins. The results show that caspases are crucial for controlling cell death and have potential as targets for treating diseases such as cancer and neurodegenerative disorders.

This summary was initially drafted using artificial intelligence, then revised and fact-checked by the author.

## Introduction

**Fig. 1:**
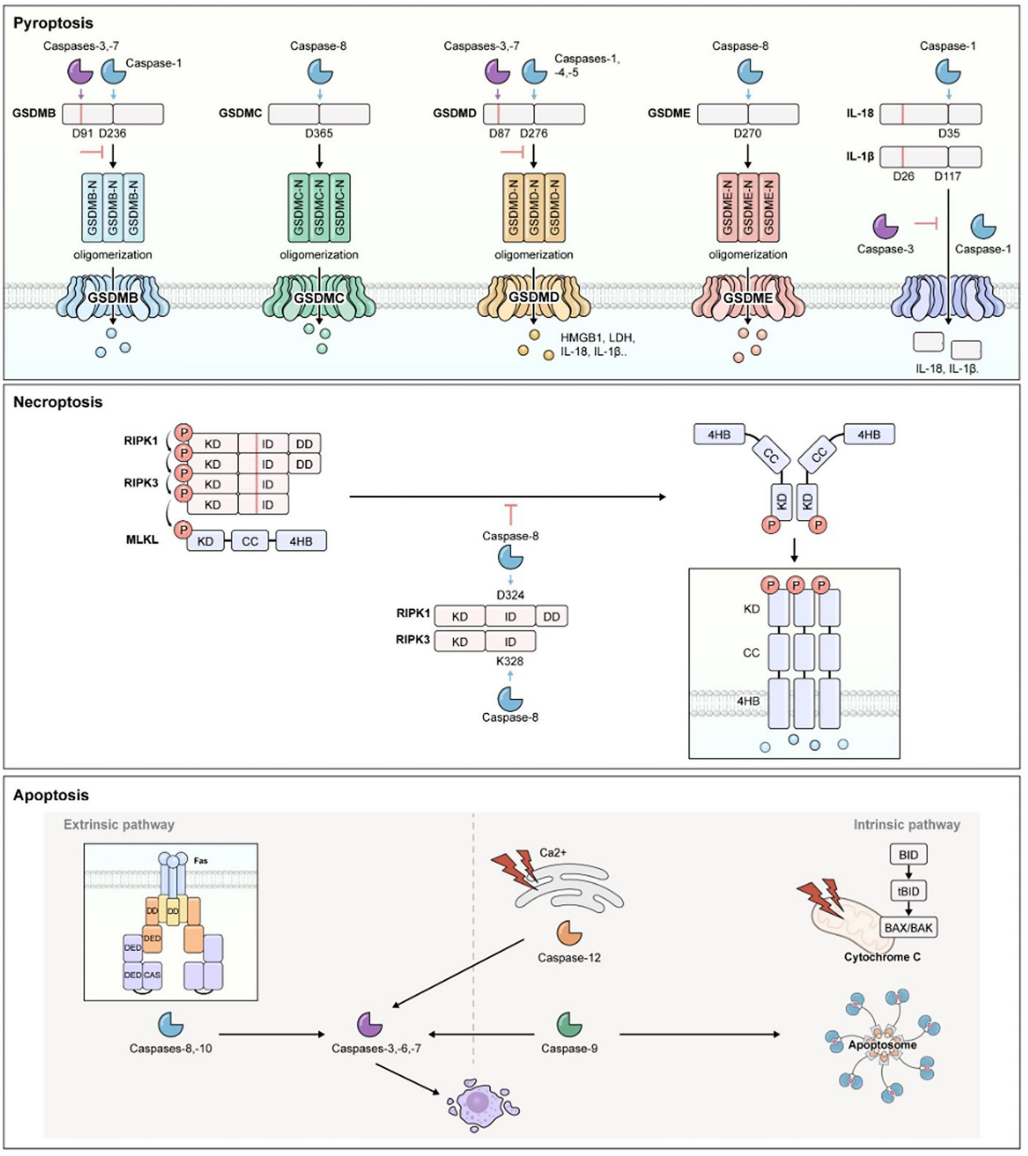
Overview of major signaling components of PCD. The molecular signaling pathways and key effector molecules involved in three major forms of PCD: pyroptosis, necroptosis and apoptosis. Pyroptosis: this process is initiated by the cleavage of GSDM by inflammatory caspases, resulting in the formation of GSDM-N, which creates pores in the plasma membrane and leads to cellular lysis. Simultaneously, pro-inflammatory cytokines such as IL-1β and IL-18 are activated by caspase-1, allowing their release through the GSDM-formed pores. These cytokines amplify immune responses, enhancing the inflammatory reaction to infections or cellular damage. However, caspases also have a regulatory role in inhibiting pyroptosis. For example, caspases-3 and -7 can cleave GSDMD at non-canonical sites, preventing pore formation. Additionally, the cleavage of GSDMB by caspases-3, -6 and -7 can suppress its pore-forming ability. Furthermore, caspase-3 can cleave IL-1β at alternative sites, inhibiting its activation and thereby reducing inflammation. This dual role of caspases in both promoting and suppressing pyroptosis underscores their crucial function in maintaining immune homeostasis. Necroptosis: initiated by the RIPK1/RIPK3-MLKL axis, RIPK3 phosphorylates MLKL, leading to its oligomerization and translocation to the plasma membrane. The incorporation of MLKL into the membrane causes rupture and subsequent cellular death. Necroptosis is tightly regulated to prevent excessive inflammation. Apoptosis: caspase-8 (extrinsic pathway) and caspase-9 (intrinsic pathway) play crucial roles in apoptosis by cleaving and activating executioner caspases, such as caspase-3 and caspase-7. These executioner caspases mediate downstream processes of apoptosis, including the dismantling of cellular structures, which leads to controlled cell death. In the intrinsic pathway, tBID translocates to the mitochondria, activating BAX and BAK, which promote MOMP. This process results in the release of apoptogenic factors from the mitochondria, ultimately leading to cell death during the execution phase of apoptosis. KD RIPK1 kinase domain, ID intermediate domain, DD death domain, CC coiled coil, 4HB 4-helical bundle, DED death effector domain, CAS catalytic subunit.

Caspases are evolutionarily conserved cysteine proteases that cleave their substrates at specific aspartic acid residues, playing a central role in programmed cell death (PCD)^1^. These enzymes are critical for maintaining cellular homeostasis and supporting processes such as development, immune responses and disease defense. PCD refers to a tightly regulated process that enables cells to undergo controlled destruction in response to developmental or pathological signals. This process is essential for organismal survival, as it prevents uncontrolled cell proliferation and removes damaged or infected cells. Caspases serve as the molecular gatekeepers of PCD, ensuring precise execution of these pathways. Among the various forms of PCD, pyroptosis, necroptosis and apoptosis are the most well-studied mechanisms, each involving distinct molecular pathways and effector molecules (Fig. 1).

Pyroptosis is primarily mediated by the gasdermin (GSDM) family, which executes this inflammatory form of cell death when activated by caspases-1, -3, -4, -5, -6, -7, -8 and other enzymes^1^. Upon activation, GSDMs release their N-terminal fragment (GSDM-N), which oligomerizes to form membrane pores. This pore formation leads to cellular swelling and lysis, accompanied by the release of inflammatory mediators, pathogen-associated molecular patterns, damage-associated molecular patterns and cytokines such as high mobility group box 1 (HMGB1), lactic acid dehydrogenase (LDH) and interleukin-1β (IL-1β)^2^ (Fig. 1). In contrast, necroptosis relies on key components such as receptor-interacting serine/threonine-protein kinase 1 (RIPK1), RIPK3, mixed lineage kinase domain-like protein (MLKL) and inactive caspase-8 (ref. ^3^) (Fig. 1). RIPK1 and RIPK3 associate to form the necrosome, which in turn phosphorylates and oligomerizes MLKL. Once phosphorylated, MLKL integrates into the plasma membrane, causing membrane rupture and necroptotic cell death. Caspase-8 modulates this pathway by cleaving RIPK1 at D324 and RIPK3 at D328, thereby disrupting necrosome assembly and inhibiting necroptosis^4^.

Unlike the lytic forms of pyroptosis and necroptosis, apoptosis is generally non-inflammatory. The extrinsic pathway is initiated by caspase-8, whereas the intrinsic pathway involves caspase-9 and mitochondria^5^. In this intrinsic pathway, caspase-cleaved truncated BH3-interacting domain death agonist (tBID) activates B cell lymphoma protein 2 (Bcl-2)-associated X (BAX) and Bcl-2 antagonist killer (BAK), which increase mitochondrial permeability and release cytochrome *c*. Cytochrome *c* then promotes the formation of the apoptosome complex with apoptotic protease activating factor-1 (Apaf-1) and pro-caspase-9, ultimately leading to the activation of caspases-3 and -7. These effector caspases cleave substrates such as poly-ADP ribose polymerase (PARP), thereby disrupting DNA repair and lamin proteins, destabilizing the nuclear envelope and cytoskeleton^6^. This cascade culminates in the formation of apoptotic bodies, a hallmark of apoptosis that facilitates orderly cell disassembly and phagocytic clearance^7^ (Fig. 1). Apoptosis was first characterized in *Caenorhabditis elegans*, where CED-3, an ortholog of human caspase-9, functions as a key caspase, underscoring the evolutionary conservation of caspases. This conservation highlights their fundamental importance in cell death regulation across species^8^.

Consequently, caspases integrate signals from multiple PCD pathways. Although ferroptosis is typically considered caspase independent, recent evidence suggests it can be inhibited by caspase-2 via stabilization of glutathione peroxidase 4 (ref. ^9^). Dysregulation of PCD can lead to diseases such as cancer, inflammatory disorders and neurodegenerative conditions. Caspases play a crucial role in the pathogenesis of these diseases through their central function in cell death regulation. A deeper understanding of caspase pathways and their modulation thus offers important therapeutic potential for disease treatments.

## PCD by caspases

### The role of caspases in PCD

**Fig. 2:**
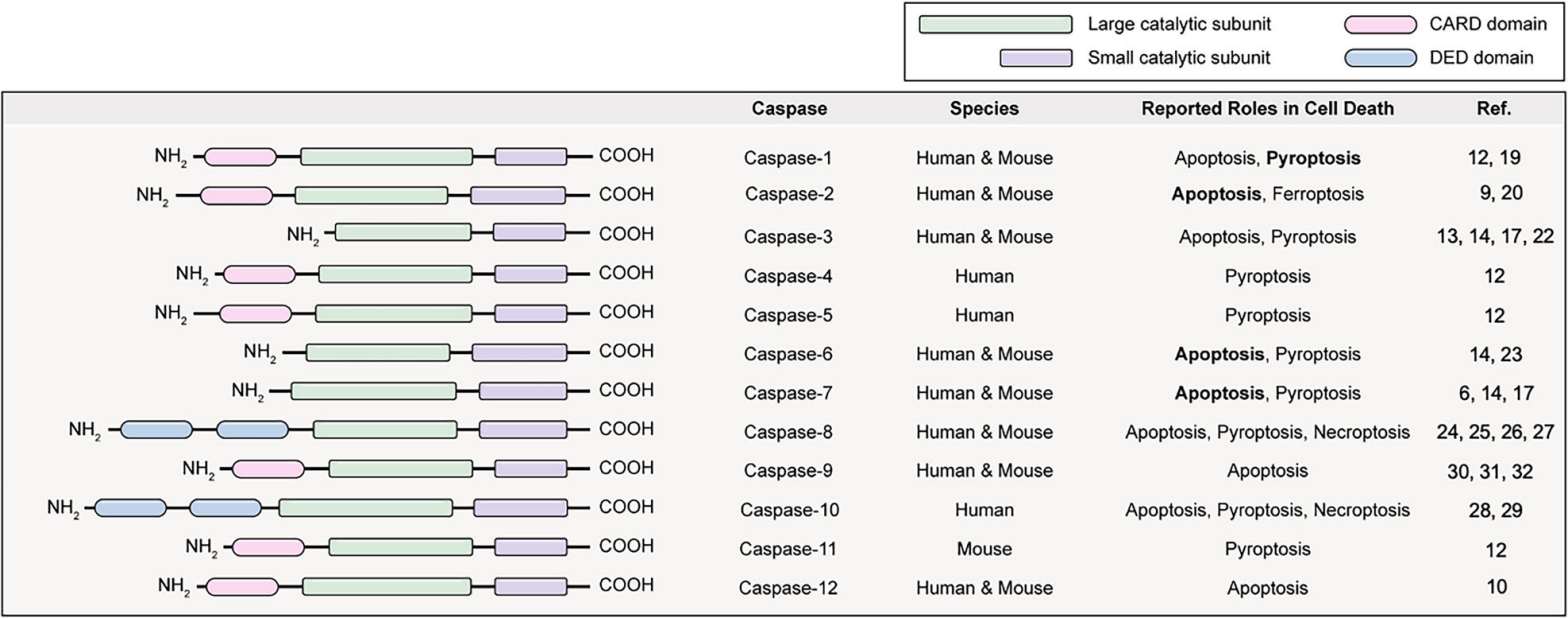
Domain structures and functional roles of caspases in PCD. The domain structures of caspases, including CARD, DED and the large and small catalytic subunits. Caspases are categorized based on their roles in apoptosis, pyroptosis, necroptosis and ferroptosis. Bold text indicates the primary cell death pathway associated with each caspase. Additionally, the table provides the mouse and human counterparts for each caspase, detailing their expression and functional roles in both species.

Caspases act as pivotal regulators in PCD, executing diverse roles across apoptosis, pyroptosis and necroptosis pathways. Caspases-2, -8, -9 and -10 primarily initiate apoptosis, while caspases-1, -3, -6 and -7 execute it^5^. Caspase-12 is involved in endoplasmic reticulum (ER) stress-induced apoptosis^10^. Additionally, caspases-1, -3, -4, -5, -6, -7, -8, -9, -10 and -11 are essential for pyroptosis. GSDMC is cleaved by caspase-8 (ref. ^11^), GSDMD by caspases-1, -4, -5 and -11 (ref. ^12^) and GSDME by caspase-3 (ref. ^13^). These cleavages facilitate pyroptosis by activating GSDMs. Necroptosis, a form of programmed necrosis, occurs when caspases-8 and -10 are inhibited (Fig. 2).

Caspases also exhibit diverse roles in pyroptosis. Typically, they cleave GSDM family, generating an N-terminal fragment that forms pores in the plasma membrane. However, the precise cleavage site depends on the stimulus and the specific caspase, determining whether the resultant GSDM-N fragment is active or inactive. An active fragment promotes pyroptosis, whereas an inactive form inhibits pyroptosis and can favor other types of cell death. Caspases-1, -3, -6 and -7 cleave GSDMB at D91 to prevent pyroptotic activation during apoptosis^14–16^. In addition, caspases-3 and -7 cleave GSDMD at non-canonical site D87, preventing its oligomerization and thereby suppressing pyroptosis^17^. Caspases-1, -2, -4, -5, -9, -11 and -12 contain a caspase activation and recruitment domain (CARD), whereas caspases-8 and -10 harbor a death effector domain (DED)^18,19^. The functions of these domains and how they interact to regulate cell death are summarized in Fig. 2.

As certain caspases are interconnected with multiple PCD pathways, shifts can occur between different PCD modes in a context-dependent manner.

### Caspases interconnected with various PCD pathways

- Caspase-1: primarily associated with inflammation-induced pyroptosis, it can induce apoptosis in the absence of GSDMD^20^.
- Caspase-2: participates in both ferroptosis and intrinsic apoptosis triggered by reactive oxygen species and ER stress^9,21^. It cleaves BID and is essential for cell cycle control and the DNA damage response.
- Caspase-3: an executioner of apoptosis associated with DNA fragmentation and mitochondrial dysfunction^22^. It also cleaves GSDMB, GSDMD and GSDME, thereby inducing pyroptosis^14,17^.
- Caspase-6: acts as an apoptosis executioner by activating caspase-8, leading to BID-dependent and cytochrome *c*-driven apoptosis^23^. It also regulates GSDMB-mediated pyroptosis^14^.
- Caspase-7: functions as an executioner of apoptosis by cleaving PARP^6^ and suppresses pyroptosis through non-canonical cleavage of GSDMB and GSDMD^17^.
- Caspase-8: plays a central role in extrinsic apoptosis and serves as a molecular switch among apoptosis, necroptosis and pyroptosis^24,25^. It activates caspase-3 and cleaves BID, cleaves GSDMC and inhibits necroptosis. Pyroptosis ensues when caspase-8-mediated extrinsic apoptosis and MLKL-mediated necroptosis are inhibited, while inactive caspase-8 contributes to the activation of caspase-1 (refs. ^7,26^). It is widely accepted that pyroptosis serves as an initial response to infectious or damaging stimuli, rather than being a consequence of apoptosis or necroptosis inhibition, making the action of caspase-8 context-dependent^27^.
- Caspase-10: involved in extrinsic apoptosis and negatively regulates caspase-8-mediated cell death^28^. Intriguingly, both caspases-8 and -10 are implicated in pyroptosis and necroptosis, as evidenced by GSDMD cleavage and MLKL phosphorylation in caspase-10 knockout and caspase-8 knockout macrophages under specific stimuli^29^.

### Caspases primarily involved in a single PCD pathway

- Caspase-4, -5, and -11: key mediators of pyroptosis as they cleave GSDMD, triggering pore formation in the plasma membrane and subsequent pyroptotic cell death^12^.
- Caspase-9: primarily associated with intrinsic apoptosis by cleaving and activating caspases-3 and -7 (ref. ^30^). Additionally, it indirectly activates GSDME by activating caspase-3 (ref. ^31^) and can inhibit necroptosis by cleaving RIPK1 (ref. ^32^).
- Caspase-12: activated by ER stress, initiating apoptosis independently of mitochondrial or membrane signals. This mechanism underscores its relevance to neurodegenerative diseases linked to ER stress and highlights the functional interplay between the ER and mitochondria^10^.

## Regulation of PCD BY caspases

### Regulating cell death through caspase domain interactions: insights from the FADDosome, RIPoptosome, inflammasome, apoptosome and PIDDosome

**Fig. 3:**
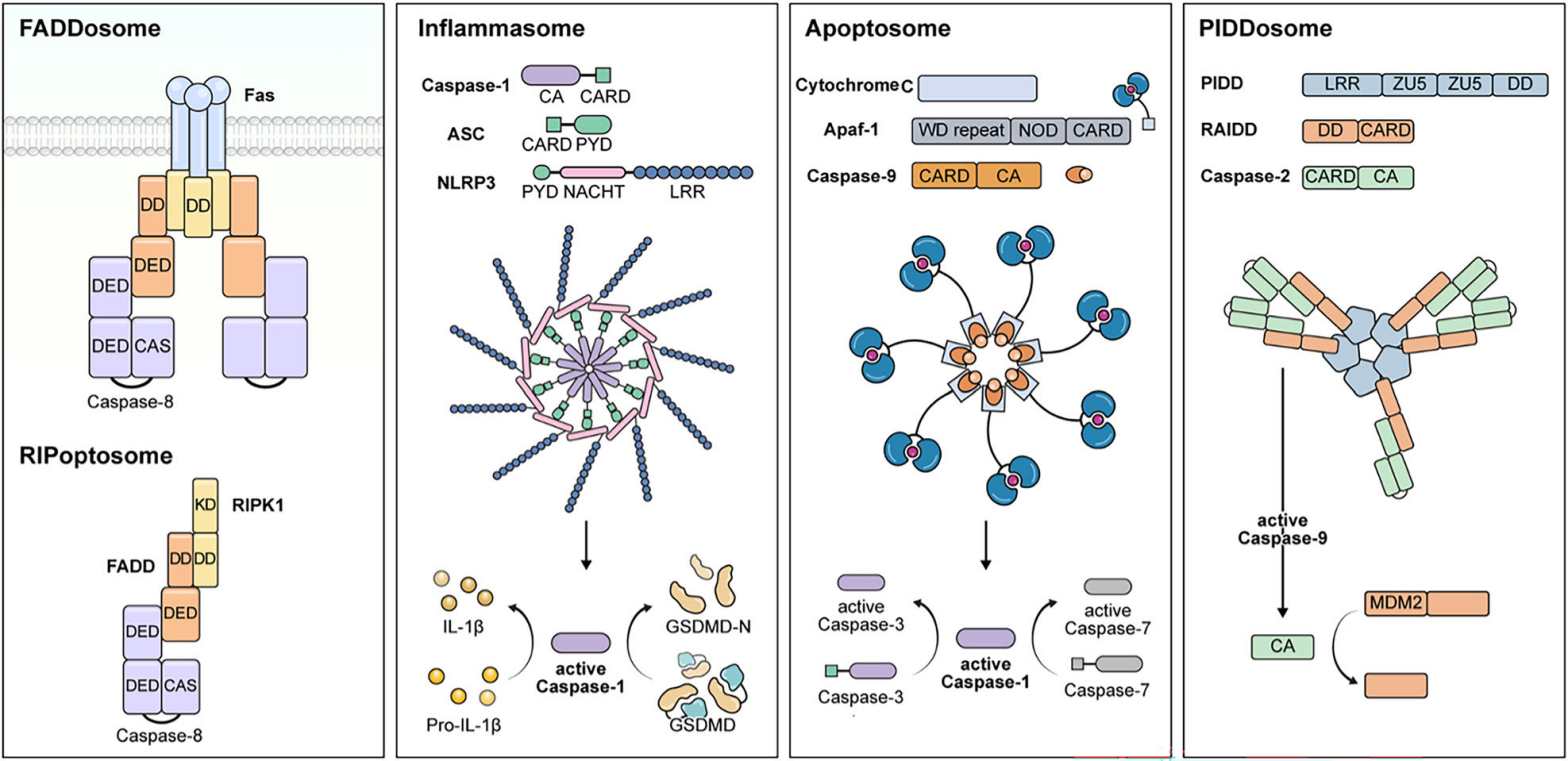
Regulating cell death through caspase domain interactions. A schematic illustrating the five major protein complexes involved in PCD pathways—FADDosome, RIPoptosome, inflammasome, apoptosome and PIDDosome. It highlights their composition and roles in activating key caspases and downstream effectors. FADDosome: composed of Fas receptor, FADD and caspase-8. The DD of Fas binds to FADD, which recruits and activates caspase-8 via its DED, thereby initiating extrinsic apoptosis. RIPoptosome: formed by RIPK1, FADD and caspase-8. The RIPK1 kinase domain (KD) and DD facilitate complex formation, leading to caspase-8 activation and subsequent apoptosis, or necroptosis if caspase-8 is inhibited. Inflammasome: a multiprotein platform consisting of NLRP3, ASC and caspase-1. The PYD of NLRP3 interacts with ASC, which in turn recruits caspase-1 via its CARD. Activated caspase-1 processes IL-1β, IL-18 and GSDMD, driving pyroptosis and inflammation. Apoptosome: consists of Apaf-1, cytochrome *c* and caspase-9. Upon release from the mitochondria, cytochrome *c* binds Apaf-1, inducing apoptosome assembly. This activates caspase-9, which subsequently activates caspases-3 and -7 to execute intrinsic apoptosis. PIDDosome: composed of PIDD, RAIDD and caspase-2. PIDD recruits RAIDD via DD interactions, which then activates caspase-2 via CARD. Caspase-2 activation modulates apoptosis and DNA damage responses by interacting with substrates such as MDM2.

Caspases are not solely catalytic enzymes; they are also regulated by interactions with specific protein domains within large multiprotein complexes. These interactions, which typically occur via CARD or DED, orchestrate critical processes such as cell death and inflammation. Various domain-mediated complexes—FADDosome, RIPoptosome, inflammasome, apoptosome and PIDDosome—govern the activation and function of initiator and executioner caspases (Fig. 3). The DED promotes binding to adapter proteins such as recruiting the Fas-associated death domain (FADD) and activating caspases-8 and -10, whereas the CARD mediates interactions with Apaf-1, apoptosis-associated speck-like protein containing a caspase recruitment domain (ASC) or RIP-associated ICH-1/CED-3 homologous protein with a death domain (RAIDD), leading to the activation of initiator caspases such as caspase-9 or caspase-1. The catalytic domain of caspases ensures selective substrate cleavage, initiating downstream signaling pathways. These intricate interactions underscore the importance of domain–domain contacts in dictating cell fate and offer substantial insights into the physiology and potential therapeutic targeting of caspase-mediated processes.

### FADDosome: caspase-8 activation in apoptosis

The FADDosome is central to the extrinsic apoptotic pathway, which is triggered by death receptors such as Fas and TNFR1. Ligand binding to Fas induces a conformational change in the receptor, thereby recruiting the FADD, which contains both a death domain (DD) and a DED. These domains are crucial for the recruitment and activation of caspases-8 and -10, forming the death-inducing signaling complex (DISC). The DED of FADD binds the DED of caspases-8 and -10, promoting their activation and subsequent cleavage of effector caspases (for example, caspases-3 and -7) to trigger apoptosis^33^. Disruption of this DED-mediated interaction impedes apoptotic signaling, enhancing cell survival.

### RIPoptosome: caspase-8 regulation in necroptosis

The RIPoptosome is a 2 MDa multiprotein complex that plays a vital role in regulating cell death^34^. It is primarily composed of caspase-8, FADD, cFLIP isoforms and RIPK1, which interact through their specific domains to mediate apoptosis and inhibit necroptosis^34^. The DEDs of caspase-8, FADD and cFLIP facilitate their recruitment into the complex, while the DD of RIPK1 allows for additional interactions^35^. In apoptotic conditions, the activation of caspase-8 within the RIPoptosome results in the cleavage of RIPK1, thus preventing necroptosis^36^. Conversely, when caspase-8 activity is inhibited, the RIPoptosome can recruit RIPK3, leading to a shift in signaling toward necroptosis^3,37^.

### Inflammasome: activation of caspases-1 and -11 in pyroptosis

The inflammasomes are multiprotein complex that activates caspase-1, a central mediator of pyroptosis. They commonly include NLR proteins (for example, NLRP3), ASC and caspase-1 (ref. ^38^). The CARD of caspase-1 binds to the CARD of ASC, whereas the pyrin domain (PYD) of ASC interacts with NLRP3, stabilizing the inflammasome^39,40^. Once recruited, caspase-1 undergoes autocatalytic cleavage, enabling the processing of GSDMD at D275, which forms plasma membrane pores and triggers pyroptosis. Caspase-1 also converts pro-IL-1β and pro-IL-18 into active cytokines. Caspase-11 participates in the non-canonical inflammasome pathway, typically activated by intracellular bacterial pathogens. Through CARD-based interactions with other inflammasome components, caspase-11 is activated and cleaves GSDMD at D275, further promoting pyroptosis and the release of inflammatory mediators.

### Apoptosome: caspase-9 activation in intrinsic apoptosis

The apoptosome is crucial for the intrinsic apoptotic pathway, typically triggered by mitochondrial dysfunction or other forms of cellular stress. It comprises cytochrome *c*, Apaf-1, caspase-9 and dATP/ATP^41^. Upon cytosolic release of cytochrome *c*, Apaf-1 undergoes a conformational change that facilitates apoptosome assembly. The CARD of caspase-9 then binds to the CARD of Apaf-1, leading to the autocatalytic activation of caspase-9. Caspase-9 subsequently activates downstream effector caspases (for example, caspases-3 and -7) to drive apoptosis^42,43^. Interference with this CARD–CARD interaction disrupts apoptosome formation and attenuates apoptosis.

### PIDDosome: caspase-2 as a negative regulator of ferroptosis

The PIDDosome, composed of p53-induced protein with a death domain (PIDD), RAIDD and caspase-2, is activated in response to DNA damage and initiates apoptosis^44^. Within this complex, the DD of PIDD interacts with the DD of RAIDD, while the CARD of RAIDD binds to the CARD of caspase-2, facilitating its activation^45^. Beyond its role in apoptosis, caspase-2 functions as a negative regulator of ferroptosis by stabilizing glutathione peroxidase 4 (GPX4), a crucial antioxidant enzyme that prevents lipid peroxidation^9^. Under oxidative stress conditions, the absence of caspase-2 leads to the destabilization of GPX4, making cells more vulnerable to ferroptotic cell death^9^. Furthermore, caspase-2 is involved in metabolic regulation, affecting lipid metabolism and mitochondrial function^46^—both of which are closely linked to ferroptosis. These findings suggest that the PIDDosome may play a broader role beyond apoptosis, integrating multiple cell death pathways to influence cell fate.

### Epigenetic regulation of caspases and its impact on cell death and disease: modulating caspase expression and the feedback loop to the epigenome

**Fig. 4:**
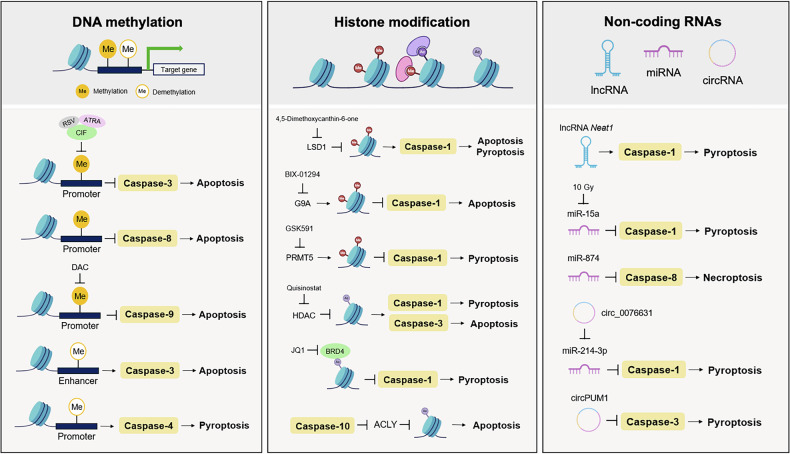
Epigenetic regulation of caspases and its impact on cell death and disease. Epigenetic regulation occurs through various epigenetic mechanisms, including DNA methylation, histone modification and ncRNAs. First, the methylation status of DNA in promoter and enhancer regions substantially impacts caspase expression. For example, promoter methylation suppresses caspase-3 and caspase-8, thereby inhibiting apoptosis, whereas demethylation of promoter and enhancer regions activates caspase-3 and caspase-9, respectively. Additionally, promoter demethylation facilitates caspase-4-mediated pyroptosis. Second, histone modifications, including methylation and acetylation of histone proteins, regulate chromatin structure and gene transcription. Various histone methyltransferases (for example, LSD1, G9A and PRMT5) modulate histone methylation, influencing caspase-1 activation. HDAC inhibitors, such as Quisinostat, promote caspase-1 activation for pyroptosis and caspase-3 for apoptosis. Furthermore, inhibition of BRD4 by JQ1 induces caspase-1-dependent pyroptosis, while histone acetylation is regulated by caspase-10 through ACLY cleavage. Third, ncRNAs such as lncRNAs, miRNAs and circRNAs also influence caspase activity. For example, lncRNAs such as Neat1 activate caspase-1 to promote pyroptosis, while circRNAs such as circPUM1 activate caspase-3. miRNAs, including miR-15a and miR-874, regulate pyroptosis through caspase-1 and necroptosis through caspase-8. Additionally, circRNAs mediate pyroptosis via the miR-214-3p/caspase-1 pathway. Dysregulation of these epigenetic mechanisms is implicated in numerous diseases, including cancer, neurodegenerative disorders and developmental abnormalities.

Epigenetic regulation of caspases involves modifications to chromatin structure and gene expression without altering the DNA sequence, thus affecting caspase expression and activity. This occurs via mechanisms such as DNA methylation, histone modification and regulation by non-coding RNAs (ncRNAs), influencing PCD and disease pathogenesis (Fig. 4).

### DNA methylation

DNA methylation, a key epigenetic modification, involves the addition of a methyl group to the C-5 position of cytosine in DNA. Generally, hypermethylation silences gene expression, whereas hypomethylation activates it. Promoter and enhancer methylation of caspases-3, -8 and -9 is linked to apoptosis, while methylation of the caspase-4 promoter is associated with pyroptosis. For instance, inhibiting DNA methylation using clofarabine, resveratrol or all-*trans* retinoic acid induces caspase-3-dependent apoptosis in chronic myeloid leukemia^47^. In contrast, DNA methylation-mediated downregulation of caspase-8 is correlated with reduced apoptosis in glioma, hepatocellular carcinoma (HCC), bladder cancer and small-cell lung carcinoma^48,49^. Moreover, systemic delivery of nanoparticle-encapsulated decitabine in a breast cancer model downregulates DNA methyltransferases 1 and 3b, leading to increased caspase-9 expression^50^. DNA hypomethylation, in combination with an activated Wnt pathway, enhances caspase-3 expression and apoptosis, potentially inhibiting intestinal tumorigenesis and offering new therapeutic insights in colorectal cancer (CRC)^51^. Hypomethylation upstream of the caspase-4 transcription start site may augment caspase-4 expression in Alzheimer’s disease, promoting amyloid-beta plaque formation by elevating IL-1β production^52^.

### Histone modification

Histone modifications, such as acetylation and methylation, regulate caspase gene expression. Acetylation of histones in promoter regions often upregulates caspase genes, whereas histone methylation can either activate or repress them, depending on the site and type of modification. Generally, methylation of histone H3 at lysines 4 (H3K4) and 36 (H3K36) correlates with active transcription, whereas methylation at H3K9, H3K27 and H4K20 is associated with transcriptional repression^53^. Histone methylation can modulate caspase-1 expression, affecting both apoptosis and pyroptosis, while histone acetylation influences caspases-1, -3 and -10, thereby impacting pyroptosis and apoptosis. Further, 4,5-dimethoxycanthin-6-one increases caspase-1 expression in glioblastoma (GBM) cells by inhibiting lysine-specific demethylase 1 (LSD1), which demethylates H3K4 and H3K9 (ref. ^54^). Blocking the histone methyltransferase G9A with BIX-01294 activates caspase-1, promoting apoptosis and reducing proliferation in non-small-cell lung cancer (NSCLC) cells^55^. GSK591, an inhibitor of protein arginine methyltransferase 5 (PRMT5), lowers H4R3me2s levels at the caspase-1 promoter, thereby elevating caspase-1 expression and inducing pyroptosis in multiple myeloma cells^56^. Quisinostat, a histone deacetylase (HDAC) inhibitor, induces both apoptosis and pyroptosis in rhabdomyosarcoma and tongue squamous cell carcinoma by modulating caspases-3 and -1 (ref. ^57^). Furthermore, inhibiting bromodomain-containing protein 4 (BRD4) with JQ1 enhances caspase-1 expression, activating the NF-κB–NLRP3–caspase-1 pyroptosis pathway in renal cell carcinoma^58^. Interestingly, caspase activity can also influence the epigenome through histone modification. For instance, caspase-10 cleaves ATP-citrate lyase (ACYL), inhibiting GCN5-mediated acetylation of histones H3 and H4 and thereby downregulating genes involved in proliferation in lung adenocarcinoma^59^.

### ncRNAs

ncRNAs are transcribed from DNA but not translated into proteins, playing key roles in regulating numerous cellular processes. Three major types of ncRNAs affect the caspase epigenome: long non-coding RNAs (lncRNAs), microRNAs (miRNAs) and circular RNAs (circRNAs).

- lncRNAs: lncRNAs interact with chromatin-modifying complexes to modulate caspase gene expression. For example, lncRNA *Neat1* stabilizes mature caspase-1, thereby promoting caspase-1 dependent pyroptosis^60^.
- miRNAs: miRNAs downregulate caspase expression by binding to the respective mRNAs and inhibiting translation. miR-15a, miR-874 and miR-214-3p affect caspases-1 and -8. High-dose radiation suppresses miR-15a, thereby elevating inflammatory cytokines and activating the caspase-1 inflammasome in colorectal carcinoma^61^. Downregulation of miR-874 promotes necroptosis by reducing caspase-8 expression^62^. miR-214-3p modulates caspase-1 in hyperglycemic environments, while circ_0076631 contributes to diabetic cardiomyopathy-related pyroptosis via the miR-214-3p/caspase-1 axis^63^.
- circRNAs: circRNAs frequently act as miRNA sponges, thereby influencing caspase expression. For instance, depletion of mitochondrial circPUM1 causes mitochondrial dysfunction and caspase-3 activation, ultimately leading to pyroptosis in esophageal squamous cell carcinoma cells^64^.

### PTM of caspases and its impact on cell death: phosphorylation, ubiquitination, SUMOylation and ADP riboxanation

Post-translational modifications (PTMs) of caspases are essential for controlling their enzymatic activity, stability, subcellular localization and interaction with other proteins. The impact of these PTMs on caspase function is increasingly recognized as a major regulatory layer in PCD pathways.

### Phosphorylation

Phosphorylation can either activate or inhibit caspase activity. For instance, p21-activated kinase 1 phosphorylates caspase-1 at S376, enhancing its activation and IL-1β production^65^. By contrast, polo-like kinase 1 phosphorylates pro-caspase-8 at Ser305, inhibiting its processing and subsequent caspase-3 activation, thereby suppressing GSDME cleavage and pyroptosis^66^. Similarly, Aurora B kinase phosphorylates caspase-2 at S384, blocking its catalytic activity and apoptotic function during mitotic stress^67^. Phosphorylation of caspase-7 at S30 and S239 by p21-activated kinase 2 hinders its activation and substrate binding, promoting cell growth and resistance to chemotherapy^68^. Akt kinase also phosphorylates caspase-9, inhibiting its activity and preventing apoptosis^69^. Notably, phosphorylation and caspase cleavage can be interdependent. Caspase cleavage sometimes exposes novel phosphorylation sites, whereas phosphorylation at cleavage sites may facilitate caspase-8-dependent substrate proteolysis^70^. Hence, phosphorylation can inhibit caspase activity under specific conditions.

### Ubiquitination

Caspase-mediated cell death pathways are tightly controlled by ubiquitination, which can either suppress or enhance caspase function, depending on the stage of cell death. For example, inhibitor of apoptosis proteins (IAPs) bind caspases-3 and -7, promoting their ubiquitination and proteasomal degradation to prevent apoptosis^71^. In the extrinsic apoptotic pathway, caspase-8 undergoes predominantly non-degradative ubiquitination, thereby inhibiting its pro-apoptotic functions^72^. The E3 ubiquitin ligase neural precursor cell expressed developmentally downregulated protein 4 (NEDD4) mediates K48-linked polyubiquitination of caspases-4 (human) and -11 (mouse), driving their degradation and attenuating pyroptosis^73^. Additionally, the Brucella effector protein TcpB promotes the ubiquitination of caspases-1, -4 and -11, leading to their degradation and reduced LPS-induced inflammasome activation^66^. Conversely, non-degradative ubiquitination can trigger caspase activation by promoting self-cleavage through specific protein–protein interactions^74^. For instance, cellular IAP1 and IAP2 catalyze K63-linked polyubiquitination of caspase-1, upregulating NLRP3 inflammasome function^75^. The linear ubiquitin assembly complex similarly attaches linear polyubiquitin to the CARD domain of caspase-1, activating it and inducing pyroptosis^66^.

### SUMOylation

SUMOylation—the attachment of small ubiquitin-like modifier (SUMO) proteins—can influence caspase localization, stability and binding to other proteins. Nuclear localization of caspase-8 correlates with SUMOylation in its N-terminal DEDs, although the precise biological outcomes of SUMOylated versus non-SUMOylated caspase-8 remain unclear^76^. SUMO-1 modification of caspase-7 may affect its nuclear localization, facilitating cleavage of nuclear targets during neuronal apoptosis^77^.

### ADP riboxanation

ADP riboxanation is an emerging bacterial modification that impairs host cell death pathways and supports bacterial replication. For example, the Shigella effector OspC3 catalyzes ADP riboxanation at Arg314 in caspase-4 and Arg310 in caspase-11, blocking their autoprocessing and preventing GSDMD cleavage, thereby inhibiting pyroptosis^78^. The *Chromobacterium violaceum* effector CopC ADP riboxanates caspases-7, -8 and -9, hindering apoptosis in host cells by interacting with calmodulin^79^.

### Subcellular localization of caspases and its impact on cell death and disease

Caspases exist as inactive pro-caspases and are activated in response to specific signals, subsequently translocating to various subcellular compartments to cleave substrates within different intracellular organelles^80^. This section discusses the subcellular localization of both pro-caspases and active caspases, emphasizing their functions within distinct cellular compartments while noting that the majority of caspases are found in the cytosol.

Mitochondria are central to multiple forms of PCD^7^. Inflammatory cell death pathways involve mitochondria through mitochondrial outer membrane permeabilization (MOMP). Caspase-cleaved GSDMs in the cytosol can target both plasma and mitochondrial membranes. Recent studies indicate that GSDMA, GSDMD and GSDME permeabilize mitochondrial membranes by interacting with cardiolipin^7,81^. However, the precise roles of mitochondrial caspases remain unclear. Pro-caspases and/or active caspases, including caspases-1, -2, -3, -4, -8 and -9, are present in mitochondria, although their exact localization within the organelle varies by cell type and apoptotic stimuli. During apoptosis, these pro-caspases are commonly released from mitochondria into the cytosol, where they become activated and initiate the apoptotic cascade. MOMP frequently marks a point of no return in cell death, regardless of caspase activation^7^.

Mitochondrial caspases may also play key roles in PCD. Caspase-2 in mitochondria is particularly important under conditions of ER stress; during infection, ER stress sensors modulate NLRP3-dependent caspase-2 signaling between the ER and mitochondria via Bid cleavage by mitochondrial caspase-2 (ref. ^82^). In addition, mitochondrial caspase-2 is critical for apoptosis triggered by oxidative stress^83^. The roles of other mitochondrial caspases, aside from caspase-2, are yet to be determined.

Caspase-12 localizes primarily to the ER membrane, where it is activated in response to ER stress, initiating stress-related and amyloid-β-induced apoptosis and synaptic toxicity in rodents^84^. Caspase-4 plays a similar role in humans as an ER stress-specific caspase and may be associated with Alzheimer’s disease pathogenesis^85^. Although pro-caspases and/or active caspases, such as caspases-7 and -9, can also be detected in the ER, their specific functions in this organelle remain unclear.

Pro-caspases and/or active caspases, including caspases-1, -2, -3, -7, -8 and -9 are present in the nucleus and can be transported there through mechanisms of active transport and passive diffusion^86^. During apoptosis, activated caspase-3 translocates to the nucleus, where it cleaves nuclear substrates, including PARP, leading to DNA fragmentation and chromatin condensation—key processes in PCD^86^. Caspase-2, characterized by a nuclear localization signal, may be present in the nucleus constitutively^87^. Additionally, SUMOylation promotes nuclear import of caspases-7 and -8, although the roles of nuclear caspases remain under investigation.

## Connecting caspases and diseases: the role of PCD

Caspases play central regulators in PCD and are intricately involved in the progression of diverse diseases, including cancer, cardiovascular conditions, neurodegenerative disorders and other inflammatory and autoimmune diseases. Regulating caspases can either drive disease progression or suppression by eliminating damaged or transformed cells, depending on the context.

**Fig. 5:**
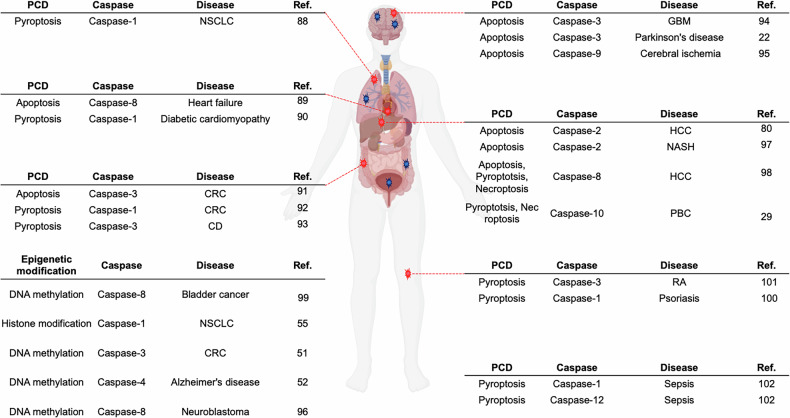
The role of caspases in PCD pathways and epigenetic regulation across diseases. The involvement of specific caspases in apoptosis, pyroptosis, necroptosis, histone modification and DNA methylation, and their associations with various diseases, along with their associations with various diseases and the corresponding affected tissues. In apoptosis, caspase-8 is associated with heart failure and HCC, while caspase-3 is linked to GBM, Parkinson’s disease and CRC. Caspase-2 is implicated in NASH and HCC, and caspase-9 is involved in cerebral ischemia. In pyroptosis, caspase-1 contributes to NSCLC, diabetic cardiomyopathy, CRC, psoriasis and sepsis. Caspase-3 is also associated with CD and RA. Additionally, caspase-8 is linked to HCC, while caspase-10 is implicated in PBC. Caspase-12 is involved in sepsis. In the context of necroptosis, caspase-8 plays a notable role in HCC, and caspase-10 is linked to necroptosis in PBC. Regarding histone modification and DNA methylation, caspase-1 mediates histone modification in NSCLC, caspase-8 is associated with DNA methylation in bladder cancer and neuroblastoma, caspase-3 is linked to DNA methylation in CRC and caspase-4 is associated with DNA methylation in Alzheimer’s disease.

Emerging evidence indicates that epigenetic modifications, including DNA methylation and histone modifications, regulate the expression of caspases in a disease-specific manner. These modifications influence their activity and contribute to the diverse roles of caspases in different pathological contexts (Fig. 5).

### Caspase and diseases: unveiling the connection

#### Lung

In NSCLC, caspase-1 drives pyroptosis through the GSDMD–NLRP3 pathway, amplifying inflammatory responses and potentially limiting tumor progression^88^. However, epigenetic silencing of caspase-1 can promote tumor cell growth in NSCLC, indicating a dual role depending on its regulation^55,88^.

### Heart

Caspase-8 regulates apoptotic pathways in cardiac diseases, contributing to disease progression. Even low levels of caspase-8-driven myocyte apoptosis have been reported to trigger the development of dilated cardiomyopathy, underscoring caspase-8 as a target for novel therapeutic strategies^89^. Additionally, caspase-1 drives pyroptosis, contributing to the development of diabetic cardiomyopathy and exacerbating myocardial ischemia/reperfusion injury through inflammatory responses and oxidative stress^90^.

### Colon

Caspase-3 and caspase-1 play critical roles in CRC progression through distinct pathways. Caspase-3 activation during chemotherapy induces apoptosis, improving treatment efficacy but also promoting tissue regeneration and cell proliferation, potentially contributing to tumor recurrence^91^. In parallel, caspase-1 drives pyroptosis via NLR family caspase activation and recruitment domain-containing 4 (NLRC4) inflammasome activation, amplifying inflammation-associated tumorigenesis^92^. Notably, DNA hypomethylation has been shown to enhance caspase-3 expression, promoting apoptosis and suppressing intestinal tumorigenesis^51^. In Crohn’s disease (CD), caspase-3 is involved in GSDME-mediated pyroptosis, promoting chronic intestinal inflammation through the release of pro-inflammatory cytokines^93^.

### Brain

Caspase-3, a key executioner caspase, is crucial in neuronal diseases. In GBM, dysregulation of caspase-3 suppresses apoptosis and promotes tumor survival^94^. Caspase-3 also plays a central role in neuronal apoptosis in Parkinson’s disease, contributing to dopaminergic neuron loss^22^. In cerebral ischemia, mitochondrial release of caspase-9 activates caspase-3, facilitating neuronal apoptosis^95^.

Epigenetic regulation underscores the diverse functional roles of caspases in neuronal diseases. In Alzheimer’s disease, DNA hypomethylation drives caspase-4 overexpression, exacerbating inflammation and amyloid-beta deposition, and contributing to neuroinflammation^52^. Similarly, promoter methylation of caspase-8 reduces its expression in neuroblastoma, contributing to tumorigenesis^96^.

### Liver

Caspase-2 is essential for maintaining liver homeostasis by regulating apoptosis and mitigating oxidative stress, thereby preventing disease progression. In HCC, caspase-2 deficiency exacerbates reactive oxygen species-induced DNA damage and genomic instability, accelerating tumorigenesis^80^. In non-alcoholic steatohepatitis (NASH), caspase-2 activation induces lipo-apoptosis by triggering ER stress, promoting disease progression^97^. Caspase-8 regulates apoptosis, pyroptosis and necroptosis in HCC, with dual roles in promoting tumor progression and preserving cellular homeostasis. Dysregulation of caspase-8 within the tumor microenvironment contributes to inflammation and drug resistance, making it a promising therapeutic target for HCC^29,98^. Caspase-10 mutations are associated with disrupted pyroptosis and necroptosis, driving the pathogenesis of primary biliary cholangitis (PBC)^29^.

### Bladder

In bladder cancer, promoter methylation of caspase-8 has been identified as a key epigenetic mechanism that reduces its expression, contributing to immune evasion and resistance to cell death, thereby driving tumor progression^99^.

### Chronic inflammatory diseases

Caspase-1 and caspase-3 play pivotal roles in the pathogenesis of chronic inflammatory diseases such as rheumatoid arthritis (RA) and psoriasis. Caspase-1 drives pyroptosis and the release of IL-18, amplifying inflammation in psoriatic lesions^100^, while caspase-3 mediates GSDME-dependent pyroptosis in RA, contributing to synovial tissue damage^101^.

### Systemic inflammatory disorders

Caspase-1 and caspase-12 exhibit opposing roles in sepsis. Caspase-1 promotes pyroptosis and amplifies inflammatory responses, contributing to the pathogenesis of sepsis. In contrast, caspase-12 functions as a negative regulator by suppressing caspase-1 activity, thereby modulating excessive immune activation^102^.

### Caspases: key molecular targets in cancer and other diseases

Caspases, which play crucial roles in cancer, autoimmune diseases and neurological disorders, underscore the importance of regulating their activity. Modulating caspase activity presents notable therapeutic potential, thus caspase inhibitors have been developed as prospective treatments for pathologies associated with cell death (Table 1). However, the development of caspase inhibitors as viable drugs poses considerable challenges. For example, Emricasan (IDN-6556) and Belnacasan (VX-765) were discontinued during phase 2 clinical trials^8^. This review aims to examine the current status of various caspase inhibitors while discussing their potential applications.

**Table 1:** Caspases: key molecular targets in cancer and other diseases.

	Inhibitor	Target caspase	Clinical status	Ref.
Pan-caspase inhibitor	Z-VAD-FMK	Caspases-1, -2, -3, -7, -8, -9 and -10 and murine caspase-11	Research use	^113,104^
	Boc-D-FMK	Caspases-3, -7 and -9	Research use	^105^
	Q-VD-Oph	Caspases-3, -7, -8 and -9	Research use	^106,114^
	Ac-FLTD-CMK	Caspases-1, -4, -5 and -11 (specific to inflammatory caspases)	Research use	^107^
	Emricasan (IDN-6556)	Caspases-3, -7 and -8	Phase 2 completed (further development discontinued). Evaluated in clinical trials for liver diseases, transplantation and NASH	^103,115^
Selective caspase inhibitor	Ac-YVAD-FMK	Caspase-1	Research use	^116^
	Mulberroside A	Caspase-1	Research use	^117^
	Chelidonic acid	Caspase-1	Research use	^118^
	Belnacasan (VX-765)	Caspases-1 and -4	Phase 2 completed (development discontinued). Evaluated in epilepsy and psoriasis	^119,120^
	M867	Caspase-3	Research use	^109^
	Z-DEVD-FMK	Caspases-2 and -3	Research use	^110^
	Ac-VDVAD-CHO	Caspases-2 and -3	Research use	^121^
	Z-IETD-FMK	Caspase-8	Research use	^122^
	Ac-IETD-CHO	Caspase-8	Research use	^123^
	Gly-Phe beta-naphthylamide	Caspase-8	Research use	^124^

### Pan-caspase inhibitor

Pan-caspase inhibitors are designed to broadly inhibit the activity of multiple caspases. Their primary clinical application is currently being investigated in the treatment of liver diseases, particularly NASH, which is characterized by excessive cell death^103^. Pan-caspase inhibitors can be classified into peptide-based and peptidomimetic inhibitors. Peptide-based inhibitors, including Z-VAD-FMK^104^, Boc-D-FMK^105^, Q-VD-Oph^106^ and Ac-FLTD-CMK^107^, have been optimized for enhanced permeability, stability and irreversible activation of caspases. These optimizations are achieved through modifications such as the incorporation of alpha-substituted ketone groups, chloro-(CMK) and fluoro-(FMK)^8^. These inhibitors have demonstrated efficacy in animal models; for instance, Z-VAD-FMK effectively reduced the percentage of peritoneal macrophages in LPS-challenged mice by promoting necroptosis and inhibiting pro-inflammatory responses^104^. Nonetheless, these inhibitors have encountered challenges in vivo due to associated toxicity^8^.

Emricasan, a prominent peptidomimetic pan-caspase inhibitor, has been investigated in clinical trials for its potential to mitigate liver injury and fibrosis by inhibiting apoptosis in hepatocytes^103^. Although preclinical and clinical studies have demonstrated its efficacy, the side effects associated with prolonged treatment ultimately led to the cessation of its clinical development. Despite its effectiveness, broad-spectrum caspase inhibitors can interfere with other crucial cellular functions, highlighting the need for selective inhibitors.

### Selective caspase inhibitor

Selective caspase inhibitors specifically target the activity of fewer than two caspase enzymes, thereby preventing their role in cell death. These inhibitors primarily focus on caspases-1, -3, -4 and -8. Substantial evidence supports the effectiveness of selective caspase inhibitors in both cellular and animal models. For instance, Belnacasan, a reversible caspase-1 inhibitor, successfully suppressed the production of IL-1β and IL-18, leading to a reduction in the inflammatory response^108^. Additionally, the caspase-3 inhibitor M867 demonstrated a reduction in vasculature and tumor progression in lung cancer cells in vitro when exposed to ionizing radiation^109^. Another caspase-3 inhibitor, Z-DEVD-FMK, exhibited neuroprotective effects in rodent models^110^. Despite advancements in safety, the development of Belnacasan was halted during phase 2 clinical trials. Caspase-1 inhibitors, which play a crucial role in pyroptosis, are recognized as effective anti-inflammatory agents; however, their application is limited due to notable teratogenic activity^108^. Currently, the majority of caspase inhibitors are utilized primarily for research purposes. Recent research has shifted its focus toward inhibitors of apoptosis^111^ and NLRP3 inhibitors^112^. Ongoing studies aim to develop more selective and viable therapeutic options, particularly for combinatorial therapies. Given the critical role of caspases across various pathways, dosage considerations may pose challenges in these combined treatment approaches.

## Discussion and perspectives

This review highlights the pivotal roles of caspases in diverse PCD pathways such as apoptosis, pyroptosis, necroptosis and ferroptosis, and underscores their importance in disease pathogenesis. We explored the intricate epigenetic mechanisms governing caspase expression, along with their specialized functions in subcellular compartments such as the mitochondria, ER and nucleus. Although many caspase inhibitors have shown promise in preclinical settings, they frequently fail in clinical trials, reflecting the complexity of precisely modulating caspase activity. Interestingly, caspase genes often cluster on specific chromosomes. Caspase-3 and caspase-6 are located on chromosome 4, while caspases-1, -4, -5, -11 and -12 cluster on chromosome 11. On chromosome 2, caspase-8 and caspase-10 lie in close proximity, suggesting that histone modifications, DNA looping or other advanced epigenetic mechanisms may coordinate their expression in tandem. As both enzymes influence apoptosis and inflammatory responses, such genomic co-localization could enable prompt and finely tuned regulation. Notably, caspase-10 appears to modulate epigenetic processes itself, hinting at a feedback loop that affects not only its own transcription but also that of nearby genes.

Subcellular localization provides an additional layer of control. Nuclear caspases that undergo SUMOylation may cleave specialized substrates, challenging the long-held view of redundancy among certain caspases. Likewise, mitochondrial caspases could target unique protein sets, highlighting potential roles in specific pathologies such as neurodegenerative or metabolic disorders. Clarifying these organelle-based distinctions calls for proteomic and interactome studies that identify substrates uniquely cleaved by caspases in each compartment. Furthermore, the three-dimensional organization of caspase gene clusters on chromosomes 2, 4 and 11 may shape their co-regulation under various physiological or pathological conditions. Chromatin conformation capture techniques (for example, Hi-C) could unravel how these loci physically interact, guiding the design of future epigenetic or gene-editing strategies to selectively modulate caspase activity. As caspase-10 can directly influence histone acetyltransferases or methyltransferases, other caspases might exhibit similar epigenetic control, creating potential new avenues for therapy in cancer and inflammatory diseases.

Such complexity underscores the need for combination approaches that leverage partial caspase inhibition alongside immunomodulatory or metabolic agents. By reducing off-target effects while preserving essential caspase functions, these dual or multipronged treatments may offer a safer and more effective route to suppress excessive cell death or inflammation. Ultimately, understanding how caspases maintain the delicate balance between cell survival and demise will be crucial for the development of innovative molecular therapies and for improving clinical outcomes in numerous diseases.
